# Aptamer and Oligonucleotide-Based Biosensors for Health Applications

**DOI:** 10.3390/bios15050277

**Published:** 2025-04-29

**Authors:** Beatriz Mayol, I. Zeina Qubbaj, Julieta Nava-Granados, Katherine Vasquez, Scott T. Keene, Juliane R. Sempionatto

**Affiliations:** 1 Department of Electrical and Computer Engineering, Rice University, Houston, TX 77005, USA; bm111@rice.edu (B.M.); jn75@rice.edu (J.N.-G.); kv35@rice.edu (K.V.); 2 Department of Materials Science and NanoEngineering, Rice University, Houston, TX 77005, USA; zq12@rice.edu

**Keywords:** aptamer biosensor, electrochemical aptasensor, health monitoring

## Abstract

Aptamers have emerged as powerful molecular recognition elements for biosensing applications, offering high specificity, stability, and adaptability. This review explores key considerations in designing aptamer-based sensors (aptasensors), with a focus on biomarker selection, aptamer design, and detection and immobilization strategies. However, challenges such as biofluid stability and reversibility must be addressed to improve biosensor performance. In this study, the potential of aptamer-based platforms in diagnostics is explored, emphasizing their advantages and future applications. Looking ahead, advances in multifunctional aptamers, integration with nanomaterials, and computational optimization are highlighted as promising directions for enhancing their effectiveness in biosensing.

## 1. Introduction

Biosensing has become one of the most critical aspects of modern healthcare, enabling early detection and monitoring of a wide range of diseases. Biosensors can give a fast response using raw samples, which makes them excellent candidates for at-home tests and fast screening of diseases [1]. In recent years, biosensors have taken the spotlight in applications such as coronavirus disease (COVID-19) tests, which helped contain the pandemic’s spread. Every work location and every home performed a fast test to detect the presence of the virus using several biofluids, such as saliva, blood, and nasal mucosa. Biosensors also play an important role in diabetes management through the development of continuous glucose sensing. Moreover, emerging technologies such as robots, autonomous vehicles, and space exploration greatly benefit from the application of sensors and biosensors. The recent advancements in biosensing platforms can be related to the fast-paced development of artificial intelligence. The power of machine learning allowed big data analysis and the discovery of new correlations and predictions using biosensing information, particularly from wearable technology. The combination of molecular and physical data augments the information given to us to prevent and detect diseases and facilitate earlier predictions for effective intervention and improved patient care [1,2].

Today, what is limiting the applications of biosensors is the biosensing technology itself. Most sensing strategies rely on enzymatic detection, where antibodies labeled with enzymes enable affinity-based recognition and signal generation. There is an urgent need to develop novel sensing technologies able to detect an extended variety of biomolecules with fast response, accuracy, and reproducibility. Naturally occurring molecules such as antibodies and enzymes can only detect a limited range of targets. Aptamers expand the range of possible targets because, despite the challenges in the SELEX method, they can be selected and designed for, in principle, any target. Additionally, in medicine, accessibility is a vital factor with preventative care relying on the ability to track biomarkers in real-time rather than relying on costly and time-consuming laboratory tests. In this context, aptamers have been presented as ideal candidates to extend biosensing applications [3]. Aptamers have emerged as groundbreaking biosensing tools that can bridge the gap between traditional antibodies and small-molecule drugs [4]. They are also known as nucleic acid antibodies and are based on short, single-stranded DNA or RNA molecules that can fold into defined three-dimensional architectures, allowing them to bind to specific targets. The high tunable affinity of aptamers is made possible due to having an infinite number of possible base pairs, facilitating a wide range of bonding possibilities [5]. They can be tailored to have high affinity, selectivity, and specificity for their targets, allowing them to differentiate between nearly identical biomarkers, such as proteins that differ by a single amino acid [4].

An aptamer’s equilibrium of folded and unfolded states is fundamental to signal detection in biosensing applications, where the equilibrium of these two states can be shifted by the presence of a target biomarker [6]. An advantage of using aptamers is their intrinsic reversibility, enabling continuous and dynamic sensing where the surface can be refreshed (regenerated), returning to their original shape after each time a molecule is detected. While both antibodies and aptamers use non-covalent interactions, aptamers can be chemically optimized to control binding strength and stability [7,8]. Another benefit of using aptamers is their ability to target multiple biomarkers at once, known as multiplexed sensing or multiplexing. This is very beneficial in healthcare applications because measuring multiple biomarkers gives the user a clearer picture of what is possibly occurring in the body. For example, these multiplexed biosensors can detect multiple antibiotics, pathogens, and cancer biomarkers in a single analysis [9].

In order to develop new aptamers, a well-defined iterative procedure is employed, known as the Systematic Evolution of Ligands by Exponential Enrichment (SELEX). The SELEX method is a way of screening that allows for the iterative selection of highly specific aptamers for a defined target [10]. SELEX starts with a large library of base pairs, which increases the likelihood of identifying either DNA or RNA sequences that can bind to the desired target. These recurring DNA or RNA motifs can assemble and create complex structures for target recognition [4]. Aptamers exist in dynamic equilibrium between their low-energy and high-energy configurations (for example, folded and unfolded). The addition of the biomolecular target, to which the aptamer has a strong affinity, lowers the enthalpy of the higher energy state, thereby driving the aptamer to change shape dynamically. This change in configuration, driven by the presence of a biomolecule, can then be leveraged to generate output signals for sensing. The equilibrium dissociation constant KD represents the extent to which the complex dissociates into a free aptamer and a free target. It will mirror the binding affinity of an aptamer to its target molecule and provide a metric for evaluating aptasensor performance [11].

Aptamers are very versatile regarding their transduction mechanisms, which allows for a wide range of sensing applications. In aptamer-based biosensors, transduction refers to the process of converting the binding of a target molecule into a detectable signal. There are different ways to extract this signal, and the technique used depends on the application that is desired. A common approach is electrochemical aptasensors (E-AB), which use a three-electrode setup with an aptamer-modified working electrode. These sensors measure electrical signals corresponding to an electrochemical reaction, usually with a redox probe tethered to one end of the immobilized aptamer. When the target molecule binds to the immobilized aptamer, the aptamer will fold, causing the redox probe (attached to one end of the aptamer) to move closer or further away from the electrode surface, therefore causing a measurable change in the signal obtained. The signal in E-AB sensors is read through the change in current–voltage characteristics (e.g., cyclic voltammetry, differential pulse voltammetry, and impedance), and the compact size of aptamers is critical in reducing the distance between the electrode and probe, allowing for high sensitivity [12].

Another strategy that can be used is fluorescent detection, where an aptamer binds to its target and undergoes a conformational change that alters the lifetime of a tethered fluorescent probe [13]. Förster Resonance Energy Transfer (FRET) and quenching are two fluorescence-based techniques that can be used to enable the detection of targets. In quenching, a decrease in signal intensity is observed when a fluorescently labeled aptamer binds to a target molecule. FRET-based biosensors use fluorescence to detect biomolecules and changes in their microenvironment [14]. FRET is a non-radiative transfer of energy from an excited donor fluorophore molecule to a nearby acceptor molecule [9]. In these biosensors, the donors and acceptors are normally fluorescent molecules, proteins, or quantum dots. When a target biomolecule is present, it will alter the distance between the donor and acceptor, resulting in a change of relative fluorescence intensity. As the distances get closer together, FRET causes the donor’s fluorescence intensity to decrease while the acceptor’s increases. This change in relative intensity will be a measurable signal that allows for the detection and quantification of a biomolecule concentration.

However, making aptasensors is not trivial. The complete process of going from a health-related disease to having a reliable biosensor for disease detection can be a long and difficult path. Starting off, selecting the correct biofluid to target, such as blood, cerebrospinal fluid (CSF), urine, sweat, or tears, can be challenging and requires careful consideration. This choice is crucial because it will determine the sensor’s environment for operation and influence which aptamer is selected. After selecting a biofluid, the SELEX process is used to identify aptamers that can specifically bind to the target in the chosen biofluid environment. Once an aptamer is selected, it often requires further modification to enhance compatibility with the chosen transduction method to ensure an efficient signal. The aptamer then needs to be functionalized in order to obtain a signal or output. Finally, it can be integrated into a functional sensor and go through validation to ensure the device works as intended in real-world conditions. After all these steps, the device can be manufactured and deployed. This review will act as a guide to navigate the process of designing and implementing aptamer biosensors.

In this review, we highlight the incredible potential of aptamers in biosensing while demonstrating the steps from start to finish on how to begin using aptamers. There is an increased need for more research in the area of aptamers, and this review is proposed to invite readers to explore the possibilities aptamers can bring. Unlike several other excellent reviews on aptamers, the following sections are intended to bring readers practical knowledge on how to start aptamer-related research. We focus on the criteria that need to be considered to select the biomarker, biofluid, and aptamer, followed by a discussion on techniques that can be employed for aptamer-based biosensors and considerations for aptamer immobilization. Finally, we show groundbreaking examples of state-of-the-art work utilizing aptamers from the inside to the outside of the body.

## 2. How to Select a Biomarker

In designing aptamer-based sensing platforms, the choice of biomarker underlies any biosensor, and it should be carefully considered because it will influence the selection of the aptamer and the overall configuration of the device. According to the FDA, biomarkers could be defined as “characteristics that are objectively measured as indicators of health, disease, or a response to an exposure or intervention, including therapeutic interventions” [15].

When selecting a biomarker, the first question that arises is whether it is clinically relevant. This means that the biomarker of interest provides meaningful insights into a patient’s health status by acting as an indicator of disease presence, progression, or treatment response [16]. Specifically, biomarkers with FDA-approved applications offer a foundation for regulatory acceptance and integration into clinical workflows [17]. Secondly, it is important to consider the accessibility of the biofluid containing the biomarker, its concentration, possible interferents, and its compatibility with the sensing platform, since it directly impacts the feasibility of biomarker detection and biosensor design.

**Figure 1:**
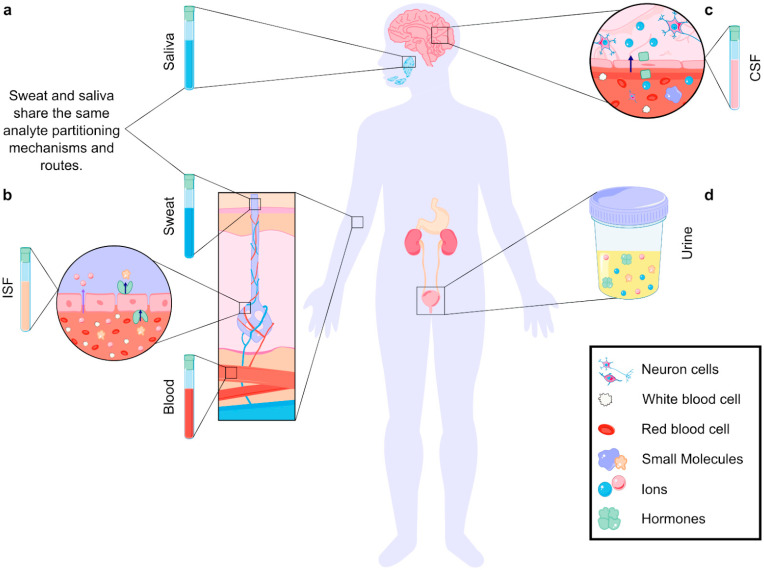
Analyte partitioning in different biofluids. (**a**) In biofluids produced by glands (sweat, saliva), analytes travel from the blood, through ISF, and finally to the glands. Analytes are filtered in each step, making the analyte concentrations in blood differ from those in the biofluids [23]. (**b**) Analytes enter biofluids through paracellular (purple arrow) and transcellular (dark blue arrow, transporter-mediated) transport. The size of the analyte, cellular structure, and partitioning mechanism define the final concentration in the fluid [23]. (**c**) Analytes can travel from the blood to the cerebrospinal fluid (CSF) through the blood–brain barrier (BBB). Partitioning mechanisms include paracellular and transcellular transport (ion transport depicted) [24,25]. (**d**) Analytes can also be found in other noninvasive biofluids, such as urine.

Biomarkers are commonly measured in biofluids such as serum, urine, saliva, blood, and cerebrospinal fluid (CSF), as well as exhaled air condensate and gastrointestinal fluids (Figure 1). Additionally, noninvasive sources such as sweat and interstitial fluid (ISF) are increasingly being explored for diagnostic purposes [18,19]. Each biofluid presents unique challenges; for example, blood and serum often contain high levels of proteins that can interfere with measurement. In contrast, sweat and interstitial fluid may require adjustments for ionic strength, pH, or composition variability [12]. Additionally, these biofluids offer the advantage of continuous monitoring with minimal discomfort, aligning with the growing demand for real-time diagnostic systems. However, real-time monitoring is not always necessary; it depends on the biomarker’s role in the disease. Biomarkers that undergo rapid changes, such as glucose or stress-related molecules, benefit significantly from dynamic monitoring [20,21,22]. Aptamer-based sensors are particularly suited for such applications due to their high specificity, fast response times, and adaptability to wearable technologies.

Aptamers can be highly sensitive to biomarker concentrations, allowing for detection over a tunable range, from micromolar to picomolar levels [26]. This sensitivity makes them particularly useful for identifying biomarkers present at very low concentrations, which is often the case in the early stages of disease progression in cancer, neurodegenerative, or cardiovascular pathologies [27]. However, because aptamers typically exhibit a single, fixed binding affinity, they tend to have a narrow range of concentrations over which they provide accurate quantification, as they saturate quickly once the target concentration exceeds a certain threshold [28]. Unlike enzymes, aptamers do not catalyze reactions that regenerate the sensing interface, so their signal is directly tied to target occupancy, making saturation a key constraint. This fact makes them less suitable for detecting biomarkers whose concentrations vary widely under different physiological or pathological conditions. To overcome this problem, aptamers can be engineered through the addition of structural modifications and biosensor design strategies, expanding their working range [29,30].

One of the advantages of aptamers is that they can target a wide range of biomarkers with high specificity, including small molecules, ions, proteins, and even whole cells, forming non-covalent bonds via van der Waals, hydrogen bonding, and electrostatic interactions [31,32]. In contrast to aptamers, antibodies cannot target molecules that do not generate an immune response, as their production relies on the activation of the immune system, which typically responds only to foreign or immunogenic substances. Small molecules, certain lipids, and other compounds often fail to elicit such a response, making antibody generation challenging without conjugation to a larger carrier protein [33].

Overall, the biomarker selection should take into account the biofluid where the target will be detected. An example of the workflow is detailed here, employing glucose, an important biomarker that can be found in all biofluids, including blood, saliva, sweat, cerebrospinal fluid (CSF), and interstitial fluid (ISF). Suitable aptamers for glucose detection are flexible structures that are able to undergo conformational changes upon binding, allowing for a signal transduction mechanism that can be integrated into a sensing platform [34]. The selectivity of the aptamer relies on its ability to recognize glucose specifically while minimizing interactions with structurally similar molecules, such as fructose or galactose, which may be present in the biofluid. Once the main features of this aptamer are established by the properties of its target, the biofluid’s intrinsic composition needs to be considered. In sweat, glucose concentrations are significantly lower than in blood [35], requiring an aptamer with high sensitivity to detect minute fluctuations accurately and remain stable at variable ionic strengths and pH conditions. In CSF, where glucose is present at approximately 60% of blood glucose levels [36], the aptamer should be optimized for a narrower dynamic range, ensuring accurate quantification without saturation. Additionally, CSF is a relatively stable and low-protein environment [37], reducing interference but requiring aptamers with high specificity to avoid cross-reactivity with other metabolic components. Conversely, for gastrointestinal fluids, the highly variable pH (ranging from the acidic conditions in the stomach to the near-neutral environment of the intestines) [38] creates the need for an aptamer that maintains its structural integrity and binding functionality across a wide pH range, potentially through the incorporation of chemically modified nucleotides or protective surface coatings.

Continuing this rationale, C-reactive protein (CRP), an important inflammatory biomarker present in biofluids such as blood, saliva, sweat, cerebrospinal fluid (CSF), and interstitial fluid (ISF), will now be exemplified. Specific biofluid characteristics remain critical. Aptamers tailored for CRP detection must demonstrate high selectivity, effectively discriminating CRP from structurally or functionally related proteins like serum amyloid A (SAA), which may be present concurrently in these biofluids. Because CRP is a large protein (114 kDa), its concentration in sweat is lower compared to blood, requiring highly sensitive aptamers with robust behavior to ionic strength and pH variations. Likewise, the same CSF and gastrointestinal fluid characteristics explained before need to be considered when designing an aptasensor for CRP. CRP is a large protein that requires from small to moderately long aptamers to reach sufficient affinity (20–80 nucleotides) [39,40]. While longer aptamers effectively recognize and bind CRP, employing redox probes within electrochemical detection systems remains feasible but presents specific challenges. For example, the electron transfer distance increases, reducing signal strength and compromising detection sensitivity. Additionally, more complex folding dynamics can lead to slower response times and higher background noise due to nonspecific interactions. In this sense, other detection techniques can also be influenced by the response time, causing issues in fluorescent intensity, aggregation when using nanoparticles, etc. However, these drawbacks can be mitigated through strategic aptamer optimization, precise surface modification, and careful control of experimental conditions.

Aside from choosing a biomarker with specific relevance for the condition of interest, considering the physiological and biochemical differences among biofluids is essential for aptamer design for sensing purposes.

## 3. How to Select an Aptamer

**Figure 2:**
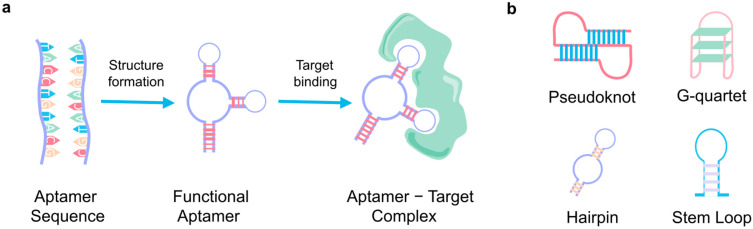
Aptamer basics. (**a**) The primary aptamer sequence, folding into its three-dimensional structure and binding with its specific target. (**b**) The four main secondary structures of aptamers.

The effectiveness of aptamer-based sensing platforms heavily depends on the ability of the aptamer to bind specifically and strongly to its target, which is determined by its secondary structure [41]. Whereas the primary structure of an aptamer is its linear sequence of nucleotides, its secondary structure refers to its three-dimensional configurations, such as stem loops, hairpins, and more complex G-quadruplex structures [42] (Figure 2). These configurations allow the aptamer to create the necessary architecture for high-affinity binding, essentially acting as a molecular “lock” that fits its target “key”. For that reason, one of the most important factors influencing the binding affinity of an aptamer is its overall shape [43]. The specific conformation of an aptamer determines how well it can fit into the binding pocket of its target, in contrast to antibodies with a rigid structure. Thus, the aforementioned non-covalent interactions, in addition to shape complementarity, contribute to the strength and specificity of the binding [44].

Particularly for small molecules, aptamers often start in a relatively unstructured or loosely folded state and undergo a significant conformational change upon binding [31]. This folding creates a precise binding pocket that tightly accommodates the small molecule, enhancing specificity. A unique feature of aptamers is their ability to distinguish between different chiral forms of a molecule, which is essential for targeting small molecules or peptides that may exist in multiple enantiomeric forms [45] and is critical across the pharmaceutical, agrochemical, food, and fragrance industries. Mirror-image molecules, despite having the same molecular formula, can exhibit markedly different biological activities, environmental behaviors, and sensory properties. This feature was explored by Challier et al. [46], who designed an electrochemical sensor capable of detecting one of the enantiomers of tyrosinamide, a small chiral molecule used as a model to exemplify the methodology. Their approach was based on the different diffusion rates between the molecule and the aptamer/molecule complex [46]. This selectivity is further enhanced by aptamers’ capacity to differentiate their target from structurally similar derivatives or analogs [47], making them extremely selective in complex biological environments, while antibodies and enzymes sometimes show cross-reactivity [48].

In the case of large molecules (i.e., proteins), aptamers generally retain more of their pre-folded secondary and tertiary structures even before binding. Rather than a complete structural reorganization, binding to a large target usually involves localized structural adjustments to optimize interactions across multiple binding sites [43,49]. Aptamers designed for proteins are key in detecting disease biomarkers, with their ability to recognize specific surface features. Due to the negatively charged phosphate backbone of aptamers, they tend to interact favorably with positively charged regions of proteins, forming stable and specific complexes [50]. Additionally, some aptamers are tailored to bind entire cells or cell surface proteins, enabling the detection of pathogens or abnormal cells [51]. For example, detecting circulating tumor cells (CTCs) in breast cancer is challenging due to their low concentration and phenotypic heterogeneity. Traditional methods, like antibody-based systems, rely on epithelial markers but may miss certain tumor cell subtypes [52]. Kolovskaya et al. explored RNA aptamers, which bind with high specificity to various breast cancer subtypes, employing magnetic separation, effectively isolating CTCs from blood samples. Due to their high affinity, structural versatility, and ability to target diverse CTCs, aptamers improved detection sensitivity in a minimally invasive manner [53]. Furthermore, Kolm et al. identified an aptamer that binds selectively to E. faecalis cells using a combined approach based on cell-SELEX and quantitative real-time PCR (polymerase chain reaction), providing a promising tool for the development of sensitive and specific detection of bacterial cells [54].

Once a target of interest is identified, selecting a suitable aptamer can follow one of three potential pathways [2]. First, a relevant aptamer may already be available in the literature, aligning well with the intended application. Therefore, the sequence of the aptamer can be ordered from a company or even synthesized in-house. Second, while an aptamer may be documented, its characteristics may make it unsuitable for the application’s specific requirements, such as sensitivity to nucleases, toxicity, and/or inappropriate size or shape. Lastly, if no aptamer is available or appropriate, a new sequence must be designed and selected through experimental processes. This aptamer selection could be performed in collaboration with researchers and specialized companies (Creative Biolabs, Novaptech, NeoVentures, AptamerLab, SomaLogic, Aptagen, Aptamer Group, Aptamer Sciences, NOXXON Pharma, etc.).

The next sections will include a description of the possibilities for aptamer selection, fabrication, and modification, as well as different strategies for their integration into sensing platforms.

### 3.1. Design, Fabrication, and Modification of Aptamers

**Figure 3:**
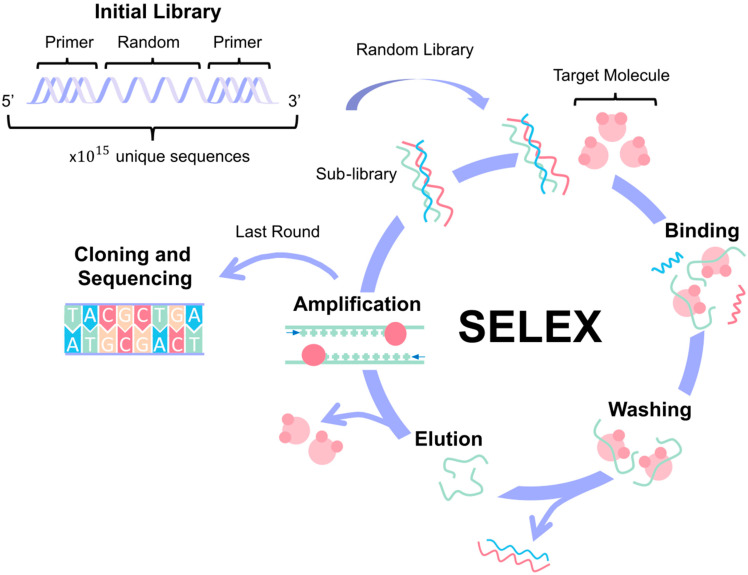
Aptamer design. The Systematic Evolution of Ligands by Exponential Enrichment (SELEX) process allows for aptamer design and selection based on the specific target molecule. It starts with the creation of an aptamer library where the sequences have a central random region flanked by constant primer regions. The process involves 1 to 16 cycles of binding, washing, elution, and amplification. In the last round, the high-affinity aptamers are identified for cloning and sequencing.

The SELEX (Systematic Evolution of Ligands by Exponential Enrichment) process (Figure 3), the most widely used method for aptamer selection, is centered around the interaction between the aptamer and its target, which forms the foundation for successful downstream applications [55,56]. Aptamers function by binding to their targets with high specificity, and this interaction can vary depending on the relative sizes of the aptamer and its target. If the aptamer is larger than its target, it tends to incorporate the target into its structure. Conversely, when the target is larger, such as proteins, the aptamer may integrate into the structure or bind to the surface [57].

The SELEX process begins with generating a diverse library of single-stranded nucleic acids, which can be either DNA or RNA. This library consists of a central random sequence flanked by constant primer regions at both the 5′ and 3′ ends. The random sequence introduces vast variability, allowing for the potential discovery of aptamers with diverse binding properties. The length of this random region, which typically ranges from 20 to 80 nucleotides, and the choice of either natural or chemically modified nucleotides are critical factors that influence the library’s diversity and functionality [57]. The next question is how to choose a library. There are established libraries available to buy (Creative Biogene, Fusion BioLabs, Aptamer Group, etc.), and it is also possible to design a customized one. When creating the initial library, researchers should consider the target’s properties (charge, hydrophobicity, and size) and the intended use of the aptamer. It is also important to maximize sequence diversity and to include any specific sequences or structural features needed [58].

Initially, the library contains around 10^15^ unique sequences, plus the target, which is immobilized onto a solid support for the SELEX procedure. The process involves several iterative rounds of selection, typically between 1 and 16 cycles, though more may be necessary for highly specific targets. Each cycle consists of four main steps: binding, where the nucleic acids interact with the target molecule; washing/elution, to remove unbound or weakly bound sequences; amplification, usually via PCR or RT-PCR, to enrich the pool of bound sequences; and sequencing, to monitor the enrichment and identify the high-affinity aptamers [59]. The main factors that affect the SELEX process are the enrichment of high-affinity binders and the elimination of low-affinity or nonspecific sequences [2].

**Figure 4:**
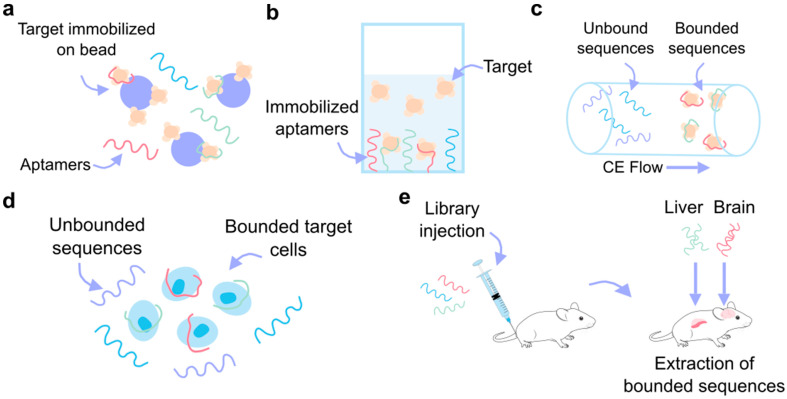
SELEX variants. (**a**) Bead-based SELEX, where the target molecule is immobilized on the beads and the aptamer remains free in solution. (**b**) Target in solution SELEX, where the aptamers are immobilized to the substrate. (**c**) Capillary electrophoresis (CE) SELEX, where the unbounded and bounded sequences are separated based on their different flow rates over the electrophoresis gel. (**d**) Cell SELEX allows for the aptamer-based recognition of specific cells. (**e**) In vivo SELEX, where the aptamer library is injected into the animal model and the bound sequences are collected from organ tissue.

Traditional SELEX variants usually involve immobilizing the target molecule on a carrier matrix while the aptamers remain in solution (e.g., bead-based SELEX (Figure 4a) and microfluidic SELEX) [55,60]. However, the immobilization of the target can introduce biases or lead to structural alterations that may impact aptamer selection. To circumvent these limitations, alternative approaches such as microarray-based SELEX [61], capture SELEX [62], atomic force microscopy SELEX [63], and particle display SELEX [64] immobilize the aptamer library instead, keeping the target in a solution, as represented in Figure 4b. Another method, capillary electrophoresis (CE) SELEX [65], keeps both the target and the library in a solution to maintain a more natural interaction between them (Figure 4c). Moreover, Figure 4d shows specialized SELEX techniques such as cell-SELEX [53] and 3D cell-SELEX [66], which allow for the selection of aptamers that can specifically recognize certain cell types or conformations. In vivo SELEX (Figure 4e) represents an even more advanced approach, where an aptamer library is injected into a living organism, and aptamers that bind to target organs are harvested, offering a more physiologically relevant context for aptamer selection [67]. A different approach to SELEX has emerged, involving the use of computational bioinformatics methods like molecular docking and molecular dynamics (MD) simulations to design aptamers for various applications. This in silico strategy can also be integrated with SELEX to improve research efficiency [68].

The structural diversity of the oligonucleotide library is a key factor that determines the success of SELEX. A library with greater diversity is more likely to contain aptamers capable of interacting effectively with a wide range of targets [2]. As mentioned before, both RNA and DNA aptamers can bind a specific target with high selectivity. Nevertheless, RNA oligonucleotides tend to offer higher structural complexity compared to DNA, which can enhance binding interactions. Conversely, DNA libraries provide certain advantages, such as increased chemical and biological stability, easier synthesis, and avoidance of the reverse transcription step necessary with RNA SELEX. Both RNA and DNA libraries have unique properties, and their structural diversity can be further increased by optimizing the ratio of different nucleotides during synthesis [2,69].

Despite advances in SELEX methodologies, several challenges remain, particularly when translating aptamer selection from controlled laboratory conditions to real-world biological environments [70]. Most aptamers are selected under highly defined buffer conditions, which often do not reflect the complexity of biological fluids. This discrepancy can lead to a limited number of aptamers performing well in actual biological contexts, such as in vivo conditions [42]. To overcome these limitations, further optimization and validation of aptamers in physiologically relevant environments are necessary, with the in vivo SELEX approach being an alternative [70].

An important aspect of this optimization process involves ensuring that the aptamer folds into its active conformation, which is essential for efficient binding. Several factors influence aptamer folding, including temperature, buffer composition, incubation time, and aptamer concentration [71]. Additionally, aptamer performance can be improved through sequence truncation, where nonessential nucleotides are removed to reduce synthesis costs and enhance binding efficiency [72]. The aptamer’s stability, binding affinity, or specificity can be increased by introducing modifications during the SELEX process or post-SELEX process [8,73], as shown in Table 1. Although these strategies could have important benefits over the aptamer performance, the cost and time investment in chemical synthesis, as well as the natural folding disruption of the aptamer, is something to consider at this stage [2].

Due to the extensive number of existing molecules, it is nearly impossible to have an aptamer for each of them. Thus, an interesting approach involves selecting aptamers for a specific feature of a family of molecules. These aptamers could be implemented in the separation, detection, or identification of similar molecules [93].

### 3.2. Getting Signal in Sensing Platforms Using Aptamers

**Figure 5:**
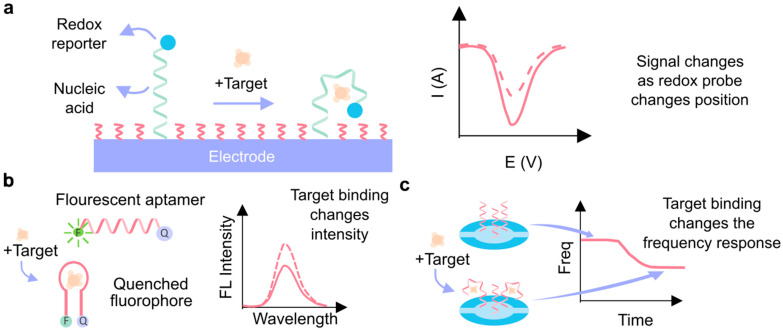
Signal transduction techniques using aptamers. (**a**) Electrochemical aptasensor. In the presence of the target molecule, the aptamer undergoes a conformational change, which is transduced by the redox reporter and measured by electrochemical techniques. (**b**) Fluorescent aptasensor. Binding with the target molecule produces a change in the optical tag (fluorophore). (**c**) Quartz crystal microbalance aptasensor, which correlates with the mass accumulated on the quartz crystal resonator with the change in its frequency [99].

The use of aptamers as biorecognition elements in biosensing, or aptasensors, is fundamentally based on the selective binding of the aptamer to its target molecule. When the target is present in a sample, it binds to the aptamer, and the binding event is transduced into a detectable signal. This signal can be generated through various physical mechanisms, depending on the biosensor format, and it is then quantified to determine the presence or concentration of the target molecule in the sample (Figure 5) [94].

Aptasensors can be configured in two primary assay formats: single-site binding and dual-site binding, depending on the size and nature of the target molecule. In single-site binding, the aptamer binds to a single target molecule, whereas dual-site binding involves aptamers interacting with two distinct sites on the target, which is particularly useful for larger biomolecules [94].

Typically, three main strategies are employed for target detection (capture, sandwich, or displacement strategies). In the capture strategy, an aptamer directly binds to the target analyte, generating a measurable signal due to a conformational change, making it suitable for simple and rapid detection [95]. It is important to consider that the aptamer needs to have a significant structure change, or it will not generate a measurable signal. The sandwich strategy employs two recognition elements: one immobilized to capture the target and another labeled to detect it, enhancing both specificity and signal amplification [96]. However, it involves a more complex assay design and requires additional reagents. In the displacement strategy, the target displaces a pre-bound molecule from the aptamer, disrupting the complex and triggering a detectable signal, which is particularly useful for competitive assays or small molecule detection, but may suffer from lower specificity if there are weak interactions between the pre-bound molecule and the aptamer, leading to nonspecific displacement. Each strategy offers distinct advantages and disadvantages depending on the target and desired sensitivity [97].

One of the unique features of aptamers is their ability to undergo conformational changes upon binding to their target molecules. These “aptamer switches” can alter their structure in response to binding, and this conformational shift can be exploited to transduce various signals (On and Off signals). For example, structural changes can generate fluorescence, colorimetric, or electrochemical signals, which can then be integrated into different biosensor platforms (Figure 5) [95]. However, converting an aptamer into a “structure-switching” biosensor poses significant challenges. The process often relies on trial and error, since established design principles for generating aptamer switches are still lacking. This makes the development of these biosensors a complex task, requiring empirical testing to optimize the aptamer’s ability to reliably produce a signal upon target binding [98].

While aptamer switches can convert binding events into measurable signals through conformational changes, their inherent signal strength is sometimes limited, which is why enzyme-assisted target recycling is employed to amplify the signal through repeated cycles of target release and rebinding. Enzyme-assisted target recycling in aptasensors leverages the catalytic activity of enzymes to repeatedly release and reuse target molecules after they bind to an aptamer. In this strategy, once the target binds to the aptamer and initiates a signal, a nuclease enzyme dissociates the target from the aptamer, allowing it to interact with another aptamer molecule. This process enhances signal amplification by enabling multiple rounds of binding events with the same target molecule, improving the sensitivity and detection limits of the aptasensor [100]. Zhan et al. [101] employed this method to develop a fluorescence aptasensor capable of detecting low levels of Aflatoxin B1, a harmful mycotoxin in agricultural products, thanks to exonuclease-assisted target recycling amplification. This method allows low detection limits (0.36 ng/mL), ensuring reliable monitoring even in trace amounts [101].

Another possible approach to prepare an aptasensor involves the design of split aptamers. Here, a specific aptamer is divided into two or more separate, non-functional fragments that can selectively reassemble in the presence of the target. However, designing split aptamers is challenging due to the need to preserve the 3D structure of the original aptamer, which can be significantly disrupted if the division occurs at an incorrect site [102]. An example of this strategy is proposed by Chang et al. [103], where a colorimetric method using split aptamers and gold nanoparticles allowed them to detect estradiol, a key estrogen hormone that, in high levels, may cause breast cancer, liver damage, hormonal imbalances, etc. They found that depending on where they were splitting the aptamer, the dynamic range and sensitivity changed, showing the importance of choosing the appropriate split fragment [103].

Regarding the signals obtained from the sensing platform, four primary types are commonly used: optical, electrical, mass-sensitive, and micromechanical (Figure 5). Table 2 provides examples of each signal type based on the employed detection strategy.

### 3.3. Immobilization Strategies and Considerations

The detection strategy used in aptasensing largely determines how the aptamer must function in the sample. For strategies that do not require the signal generated by the aptamer to be localized close to the sensing platform, such as in most fluorescence, colorimetric, and chemiluminescence techniques, aptamers are most commonly employed free in solution. However, in situations involving flow and sensing techniques that benefit from having the aptamer-generated signal close to the sensing platform, particularly for enhancing signal readouts (e.g., electrochemical, mass-sensitive, and micromechanical techniques), aptamers are typically immobilized on the sensing platforms. Because aptamer proximity is key in these approaches, aptamer length must be taken into consideration. Although shorter aptamers increase signal proximity to the sensing platform, longer aptamers may be required for larger bioanalytes to allow for proper aptamer folding. Nanoparticles are also beneficial in immobilization contexts, as they can impart aptamers with biochemical capabilities that enhance detection by the sensing platform and increase the surface area for aptamer immobilization.

**Figure 6:**
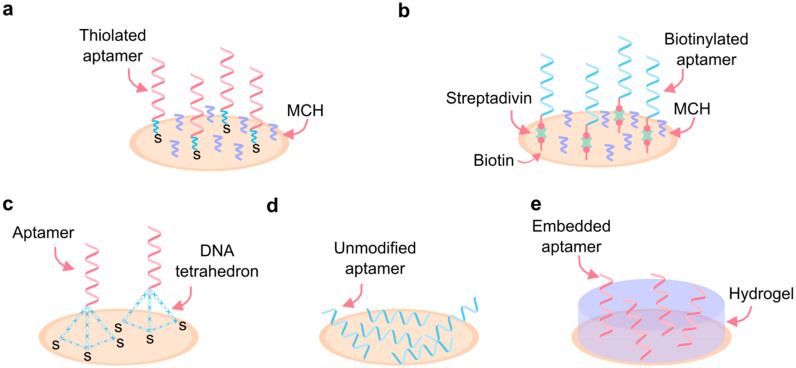
Immobilization of aptamers. (**a**) High-density covalent binding to the surface by thiol-metal interaction. (**b**) High-density affinity-based binding by streptavidin/avidin-biotin interactions. (**c**) Low-density DNA tetrahedron aptamer surface binding. (**d**) Adsorption binding of unmodified aptamers. (**e**) Immobilization by embedding aptamers in a hydrogel matrix. MCH: 6-Mercapto-1-hexanol.

The specific method of aptamer immobilization significantly impacts their sensitivity, stability, and accessibility [133,134]. Consequently, various immobilization techniques have been developed to achieve optimal results, with careful consideration given to the target analyte and biofluid when selecting an aptamer immobilization strategy. Below, we review different approaches to this process (Figure 6).

For analytes present at high concentrations in a target biofluid, a common immobilization method involves the covalent or affinity-based binding of aptamers to the surface of interest (e.g., an electrode or nanoparticle). Although covalent and affinity-based bonds can make aptamers more rigid and less flexible, high analyte concentrations in biofluids can help counteract the resulting sensitivity reduction. The relative concentration of an analyte depends on its physiological concentration range and the biosensor’s detection limits. Analytes, such as glucose, which are present in high quantities under physiological conditions and can easily fall within a biosensor’s detection range, may be considered high-concentration analytes.

One widely used technique relies on thiol–metal interactions, where aptamers are either directly labeled with a thiol group or attached via a thiol-containing alkyl chain or poly(thymine) linker. The thiol groups interact with the metal, usually gold, on the electrode surface, forming a covalent bond [138,139]. Streptavidin/avidin–biotin interactions, a common affinity-based binding technique, involve tethering biotin-labeled aptamers to avidin-modified surfaces and provide similar aptamer properties to covalent bonding techniques [140]. Specifically, these methods offer strong, stable aptamer attachment to surfaces.

One limitation of this technique is the nonspecific adsorption of aptamers onto the surface due to electrostatic or hydrophobic interactions, which reduces sensor sensitivity by altering aptamer conformation. Backfilling, which involves introducing mercaptohexanol (MCH) either after or alongside the aptamers, helps reduce nonspecific adsorption by blocking unoccupied spaces on the electrode surface [141,142].

For analytes present in low concentrations in biofluids, detection may benefit from aptamer immobilization via adsorption. Adsorption utilizes the negatively charged DNA backbone to adhere aptamers to positively charged surfaces. This technique does not require aptamer modification, making it the simplest of all methods. Maintaining aptamers in their native state also makes them particularly desirable for low-concentration analytes, as their ability to fold easily makes them highly sensitive [142]. However, this method may not be ideal in scenarios involving fluid flow, as aptamers can easily become displaced by moving fluid. In such cases, covalent or affinity-based immobilization techniques may be more appropriate [143].

For large bioanalytes, the use of DNA nanostructures can be advantageous. In this approach, aptamers are tethered in controlled quantities to DNA-based tetrahedrons, which can be fabricated in various sizes depending on the analyte’s dimensions. These structures are then placed on the surface, allowing for precise spacing of aptamers [144]. In addition to providing adequate spacing for the binding of large bioanalytes, the precise control offered by DNA tetrahedrons can reduce steric hindrance and electrostatic repulsion, which can occur when aptamers are too closely spaced [145].

In biofluids with harsh conditions, such as extreme pH or high salt concentrations, embedding aptamers in a matrix can enhance their stability and maintain performance. In this technique, matrices such as hydrogels and polymeric films create ideal conditions for aptamers. Although this approach limits the degrees of freedom of the aptamers, it enhances their stability under harsh conditions while preserving their performance [146].

An important consideration in biofluids is the presence of other biological molecules, such as proteins, amino acids, and nucleic acids. These molecules can adhere to the electrode surface and cause “biofouling,” which impedes the binding of the target analyte. While few techniques exist to address this challenge, some methods used for aptamers include zwitterionic peptides, aromatic thiols, and antifouling polymeric coatings [147,148].

In all these techniques, various factors during the aptamer immobilization process can be adjusted to optimize their function and immobilization. Key parameters include the ionic strength of the buffer used; pH; and aptamer incubation time, concentration, and conformation (open or closed) [149].

## 4. Challenges and Opportunities in the Use of Aptamers in Sensors

Aptamers, as nucleic acid molecules with high specificity and affinity for their targets, offer significant potential in sensing applications. Their advantages include ease of generation, low manufacturing costs, minimal batch-to-batch variation, reversible folding properties, and low immunogenicity when compared to monoclonal antibodies [31,150].

Despite these benefits, the development and application of aptamers face significant challenges. The SELEX process is often labor-intensive and inefficient. This method requires multiple cycles of selection and amplification to enrich high-affinity aptamers, which can introduce biases, such as the loss of rare binding sequences and unintended amplification of non-target sequences. These issues can compromise the quality and binding affinity of the selected aptamers [151]. Researchers are making efforts to refine the selection protocols, optimize library design, and incorporate new analytical and computational techniques, thereby overcoming SELEX’s inherent challenges while enhancing the specificity and efficiency of aptamer generation [152].

Another critical limitation is the performance disparity between in vitro and in vivo conditions. Aptamers selected in vitro may fail to function effectively in biological systems due to their intrinsic physicochemical properties, such as ionic strength, pH, and the presence of competing molecules [151]. Additionally, they may lose their structure and binding capability under extreme temperatures, limiting their usability in diverse sensing environments [153]. Therefore, in vivo applications depend on animal models to generate aptamers compatible with complex biological environments, along with chemical modifications to improve resistance to nuclease degradation and prolong action times [31,70]. All these facts, while improving stability and functionality, increase the complexity and cost of aptamer development. Regarding the reversibility of aptasensors, incomplete release of target molecules during regeneration leaves binding sites partially occupied. This residual occupancy reduces the sensor’s sensitivity and performance over repeated use cycles [154]. Efforts are underway to develop smart regeneration strategies using electrical or chemical cues to reset the aptamer conformation after target binding.

Despite these challenges, aptamers also present significant opportunities in biosensor development. Their synthetic nature allows for high batch-to-batch reproducibility and cost-effective production [155]. Their ability to undergo structural modifications enables fine-tuning for improved affinity and specificity [150]. Moreover, aptamers can be integrated into different sensing platforms, expanding their versatility. The potential for real-time point-of-care, wearable, and implantable applications further highlights their growing relevance in diagnostics and prognostication of diseases.

## 5. Diagnostic Applications of Aptamers

Aptamers have revolutionized biosensing by enabling the rapid and real-time detection of biomarkers for a wide range of diagnostic applications. Their reversibility, tunable concentration range, versatility in selecting targets, and stability have shown they are well suited for being applied in the real world. An aptamer’s ability to respond dynamically to changes in biomarker concentrations allows for continuous monitoring, a key aspect in pharmacokinetics, drug delivery, in vitro metabolite detection, and therapeutic drug monitoring. This is also important for neurological applications, where going through the blood–brain barrier (BBB) is a major challenge. The BBB is extremely selective and restricts the entry of many molecules, making delivering pharmaceuticals a huge challenge. Aptamers have become an increasingly useful tool for being able to target very specific molecules and diffuse passively into the BBB due to their small size. Additionally, the use of aptamer-based biosensors in point-of-care (POC) diagnostics and wearable technologies enables noninvasive, high-sensitivity detection of biomarkers, paving the way for improved disease monitoring and personalized medicine.

### 5.1. Integrated and Point-of-Care Aptasensors

**Figure 7:**
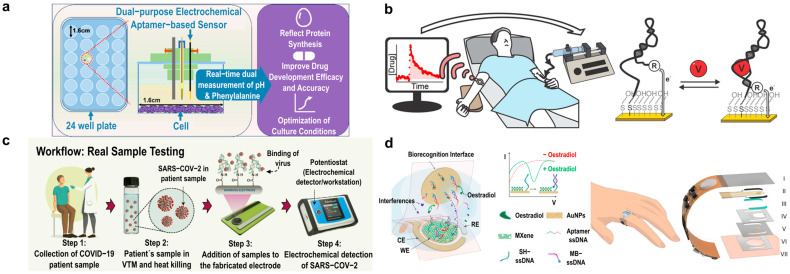
Diagnostic applications of aptamers. (**a**) Electrochemical aptamer-based sensor for pH and metabolite monitoring in cell culture media for up to 72 h in multiple cell lines. Adapted from [155]. (**b**) Therapeutic drug monitoring with electrochemical aptamer-based sensor for vancomycin detection. Adapted from [157]. (**c**) Point-of-care electrochemical aptamer-based sensor for COVID-19 detection. Adapted from [158]. (**d**) Skin-interfaced electrochemical aptamer-based sensor for picomolar detection of oestradiol in sweat. Adapted from [126].

One of the many ways aptamer-based biosensors are being explored is for the in vitro monitoring of metabolites. Metabolites are usually measured using techniques like mass spectroscopy, nuclear magnetic resonance, and different electrochemical and optical methods. These techniques, however, require the sample to be extracted, prepared, and analyzed, making real-time measurement not possible. The traditional metabolite monitoring techniques, like those mentioned, often lack time resolution and are limited to single-time-point and single-parameter measurements, making it difficult to track dynamic metabolic changes. Using aptamers allows for the use of multiplexing and reversible sensing as an advantage over current techniques. Yiling Yang et al. [156] developed a dual-purpose electrochemical aptamer-based biosensor designed for tracking pH and metabolite concentrations in cell culture media over a period of time (Figure 7a). This biosensor was able to continuously monitor and track the pH through the shift in redox probe potential to see changes in the cell culture environment and phenylalanine levels to track metabolic activity. Silver ion cytotoxicity is a concern for many continuous cell culture monitoring systems. Ag/AgCl reference electrodes can cause silver ions to go into the medium and be toxic to cells, disrupting activity. There are some ways to help this issue, such as encapsulation to mitigate this toxicity (which can help slow down the diffusion of silver ions, using alternative reference electrodes that are non-toxic), or constant medium changes (which can be timely and costly). To address this issue, the researchers developed a leak-free electrode to mitigate silver ion cytotoxicity, ensuring long-term stability and accuracy. Using these sensors, the pH and phenylalanine levels were successfully monitored for 72 h in multiple cell lines, showing the potential for real-time and continuous metabolic tracking [156].

E-AB sensors are being developed for therapeutic drug monitoring (TDM) and high-precision controlled drug delivery. Philippe Dauphin-Ducharme et al. [157] presented an E-AB sensor designed for vancomycin, a drug requiring tight dosing control (Figure 7b). Vancomycin is used to treat bacterial infections; however, it has a very narrow therapeutic window, meaning that if someone is in the wrong range, it can be toxic to the kidneys and cause hearing damage. Current TDM methods are often utilized through blood sampling in a lab, which can have slow results and lack real-time measurement. This device was able to demonstrate the measurement of vancomycin in finger-prick samples, which is quicker and still very precise. The results demonstrated the potential for personalized, feedback-controlled drug administration directly in blood and in vivo, as they were able to enable the quick single-step detection of specific molecules [157].

Aptamers also have shown promise for point-of-care (POC) and at-home diagnostics. A notable example is their use in COVID-19 detection, where aptamers have gained increasing attention as an alternative to antibodies. Narlawar Shrikrishna et al. [158] developed a screen-printed carbon electrode-based electrochemical aptasensor that demonstrated reliable detection of the virus in various environments, offering a fast and effective diagnostic tool for widespread use (Figure 7c). Using SELEX, the receptor binding domain of SARS-CoV-2 was selected as the target, making this a highly specific aptamer. This aptasensor offers a rapid and cost-effective device while highlighting the potential of aptamers for POC applications [158].

Aptamer-based biosensors are also being explored for diabetes management technologies, such as for glucose and insulin detection. There are many different devices on the market for diabetes management, but this aptamer sensing strategy shows a way to capture important biomarkers, such as insulin resistance, for a quicker diagnosis process. Jihong Wang et al. [159] presented a surface-enhanced Raman scattering aptasensor that uses gold bipyramidal nanoparticles to enhance the sensitivity of the signal. This sensor can detect multiple biomarkers related to diabetes, like insulin, which can also be used to differentiate type 1 from type 2 diabetes. The highly selective sensor can detect the changes in molecules with the presence of the aptamer, and these structural changes can indicate glucose or insulin binding. The researchers were also able to validate their testing with saliva samples and showed that it can be a noninvasive technology. This precise simultaneous monitoring of both glucose and insulin is important and has become a widespread tool for diagnosis [159].

### 5.2. Implantable Aptasensors

Another area of focus has been the development of implantable sensors for continuous pharmacokinetic analysis. Pharmacokinetic analysis is vital for seeing how drugs move in the body in order to ensure correct dosing is given to avoid toxicity. A common issue for many pharmacokinetic models is the lack of real-time measurements, which often rely on approximations. Matthew McDonough et al. [160] showed the ability of high-resolution temporal datasets to model dynamic pharmacokinetic profiles with strong statistical reliability. They achieved this by using E-AB sensors to monitor plasma concentrations of tobramycin in rats and statistically compared this to traditional one- and two-compartment pharmacokinetic models, therefore also understanding the impact of kidney function. Using these sensors allowed for rapid results and the ability to monitor hundreds of data points at once, and after monitoring the drug elimination rates, it was found that kidney function significantly affected the rate of tobramycin elimination [160].

Similarly, monitoring drug levels in real-time is crucial for the diagnosis and management of drug intoxication and overdose, where traditional methods rely on blood draws and do not provide immediate results. Obtin Alkhamis et al. [161] developed two cocaine detection sensors: an in vitro fluorescent sensor and an electrochemical aptamer-based sensor designed for real-time measurements in vivo. The first sensor was a fluorescent sensor that detected cocaine in vitro, and the second was an E-AB sensor that allows for real-time and high-frequency cocaine monitoring in the circulating blood streams of live animals. They also developed new aptamers through SELEX that could specifically bind to cocaine with stronger binding than previous aptamers, allowing for improved cocaine detection for both in vivo and in vitro environments, offering potential applications for clinical diagnostics and pharmacology research [161].

Aptamers have also been increasingly used for neuropharmacology, particularly for tracking drug transport across the BBB and monitoring neurotransmitter levels. Their specificity and high affinity are a huge advantage when it comes to working with such complicated systems as the brain, where highly specific targeting is required. Additionally, the small size of aptamers makes them ideal for drug transport through the BBB. Julian Gerson et al. utilized E-AB sensors to measure vancomycin transport across the BBB in real-time, achieving temporal resolution on the order of seconds. They focused on tracking the movement of the drug from the injection site in the veins, measuring drug concentrations in both locations, allowing for spatial resolution [162].

Neurotransmitter monitoring is essential for understanding neurological disorders as well as natural brain function. Chuanzhen Zhao et al. [163] developed an implantable aptamer-field-effect transistor (FET) neuroprobe for in vivo neurotransmitter monitoring of serotonin. These neuroprobes were fabricated using microelectromechanical system technology and tested in simulated tissue matrices like brain tissue and under electrical stimulation to mimic the release of serotonin. The FET neuroprobes allow for a high spatial resolution since they can bind directly and do so in seconds, allowing them to monitor neurotransmitter changes in real time. The sensors demonstrated functionality across different testing environments, such as stimulated brain tissue and electrical stimulation, which shows their potential for continuous neurotransmitter monitoring in neural research and for medical diagnostics [163].

### 5.3. Wearable Aptasensors

Aptamers have emerged as a game-changing tool for wearable biosensors, expanding the range of detectable analytes and improving the feasibility of continuous monitoring. Unlike antibodies, which have been widely used in biosensing, aptamers offer key advantages for wearable applications due to their stability, ease of modification, and ability to undergo reversible binding, making them ideal for continuous, real-time monitoring. Enzyme-based biosensors have been the cornerstone of wearable sensing, but the number of analytes detectable by enzymes is limited to a few small metabolites. The introduction of aptamers has introduced groundbreaking possibilities, allowing for the sensing of a broader range of biomarkers, including hormones, cytokines involved in immune response, stress, and metabolic health. Aptamers have shown great promise in monitoring analytes in noninvasive biofluids for wearable applications [164].

Ye, Cui et al. [126] reported a significant advance in the field by developing a wearable aptamer nanobiosensor for noninvasive monitoring of female hormones from sweat (Figure 7d). This study’s innovation lies in its ability to measure hormones like estrogen with high sensitivity and specificity, which were previously difficult to monitor continuously and noninvasively. By addressing the challenge of wearable biosensors for hormone detection, this sensor enables real-time reproductive health tracking, providing valuable insights for fertility management and hormone-related disorders [126].

Aptamers have also allowed for the study of other hormones in noninvasive biofluids such as cortisol. Cortisol regulates metabolism and most of the stress and immune response. Continuous tracking of cortisol in noninvasive biofluids, such as sweat and interstitial fluid, is crucial for understanding and managing stress-related health conditions. Unlike traditional blood-based tests, which provide only a snapshot of cortisol levels at a single point in time, continuous monitoring allows for real-time tracking of diurnal patterns and acute stress responses. This is particularly important for detecting chronic stress, adrenal disorders, and mental health conditions such as depression and anxiety.

Recent studies have demonstrated the unique capabilities of aptamer-based wearable cortisol sensors, showcasing both the versatility and the technical advantages of aptamer technology. Singh et al. [165] developed a noninvasive, wearable electrochemical sensor for real-time cortisol monitoring using a pseudoknot-assisted aptamer. A pseudoknot is a complex secondary structure formed by folding the aptamer into interlocking loops, which enhances the binding stability and reduces background noise, improving signal clarity. The sensor integrated a flexible microfluidic system, which allowed for the separation of old and new sweat, ensuring that the cortisol readings were accurate and reflective of real-time physiological changes. This work would not have been possible without aptamers, as antibody-based systems lack the ability to regenerate signals and maintain specificity over prolonged periods in complex biological fluids like sweat [165].

Wang et al. [166] introduced an innovative field-effect transistor (FET) sensor platform for wearable cortisol monitoring, where the binding of cortisol to an aptamer immobilized on a thin film of indium oxide (In_2_O_3_) produced a measurable electrical signal. FET sensors work by detecting changes in the electrical field at the sensor surface, which alters the conductivity of the material. With this approach, electrochemical detection is possible without the need to use a redox probe. The sensor was incorporated into a smartwatch, enabling continuous and wireless monitoring of cortisol levels. The FET configuration allowed the sensor to be highly sensitive and low-power, making it ideal for long-term use in wearable devices [166].

Aptamers have also made a great impact in interstitial fluid analysis. Interstitial fluid (ISF) has emerged as a highly valuable biofluid for wearable biosensors due to its rich composition of biomarkers that reflect physiological and pathological states. ISF surrounds the cells in tissues and serves as a transport medium for nutrients, waste products, hormones, electrolytes, and signaling molecules. ISF can be accessed noninvasively through microneedle technology, offering a promising alternative for continuous health monitoring without the discomfort and complexity of blood sampling. Wearable biosensors that sample ISF can provide real-time insights into metabolic status, drug pharmacokinetics, immune responses, and electrolyte balance, opening the door to personalized medicine and early disease detection. However, the dynamic and complex nature of ISF presents significant technical challenges for biosensing, including low biomarker concentrations, signal interference from complex biological matrices, and sensor stability over time. Aptamers have addressed many of these challenges, making continuous ISF microneedle-based analysis feasible for the first time. Microneedles provide a minimally invasive route for accessing biomarkers in the dermal layer while maintaining high user comfort and compliance.

Wu et al. [167] developed a microneedle-based aptamer sensor for continuous therapeutic drug monitoring in ISF. Therapeutic drug monitoring (TDM) is critical for optimizing drug dosing, reducing side effects, and improving treatment outcomes. The sensor used aptamers specific to the target drug molecules, allowing for real-time pharmacokinetic tracking with high sensitivity and specificity. The ability to track drug concentration in real time represents a significant advancement for precision medicine, allowing clinicians to adjust dosing dynamically based on individual patient responses [167].

Friedel et al. [168] presented a microneedle-based electrochemical aptamer sensor used for real-time molecular monitoring of phenylalanine, a biomarker clinically important for managing phenylketonuria (PKU)—a metabolic disorder where elevated phenylalanine levels can cause neurological damage if untreated. The use of aptamers provided high specificity and reversible binding, allowing for continuous, real-time monitoring without sensor degradation. The sensor achieved consistent signal detection over 90 days of dry storage and a clinically relevant lag time (~20 min), reinforcing the potential of aptamers for pharmacokinetics and chronic disease management [168].

The use of aptamer-based microneedles opened several opportunities for tracking molecules in ISF. The study by Zheng et al. [169] presented an innovative hydrogel-based microneedle patch for the extraction and analysis of interstitial fluid (ISF) biomarkers. The hydrogel microneedles swell upon skin insertion, facilitating efficient ISF collection for ex vivo analysis. The biosensor demonstrated the ability to detect four key biomarkers using aptamers: glucose (for diabetes monitoring), ATP (for cellular energy balance), L-tyrosinamide (a metabolic and cancer biomarker), and thrombin (for blood coagulation). The aptamer-based strand displacement strategy allowed for reagentless detection, where target binding induced fluorescence by separating the quencher from the fluorophore. This approach achieved high sensitivity and specificity, thanks to the strong thermodynamic stability of aptamer-target binding. The use of aptamers allowed for reversible, specific binding and signal generation without post-processing steps, making the system highly suitable for continuous health monitoring. The combination of hydrogel microneedles and aptamer-based detection represents a significant advance in noninvasive biomarker monitoring, particularly for metabolic and hematologic health tracking [169].

The opportunities unlocked by aptamers for wearable biosensors are substantial. Their ability to target a wide range of biomarkers means that future wearables could monitor not only metabolic health but also immune response, mental health, and even early-stage cancer markers. Aptamers’ small size and ease of synthesis allow for multiplexed sensing platforms, where a single wearable could monitor dozens of biomarkers simultaneously. This multiplexing capability could lead to a new generation of personalized health devices, providing continuous, real-time insights into an individual’s physiological state [170]. The development of flexible, low-power electronics and improved signal processing algorithms will further enhance the utility and accuracy of aptamer-based wearables. By overcoming current challenges, aptamers have the potential to transform wearable biosensing from single-analyte monitoring to comprehensive, dynamic health tracking, unlocking new possibilities for early disease detection, chronic disease management, and personalized medicine.

## 6. Outlook and Future Directions

As aptamer-based technologies continue to advance, efforts are focused on addressing key challenges to improve their efficiency, stability, and versatility in sensing applications. The current limitations of the SELEX process, including its high cost and low yield, are driving the development of more efficient fabrication strategies. Future research is likely to benefit from computational tools that predict aptamer-target interactions, reducing the reliance on time-consuming trial and error and large combinatorial libraries. In parallel, integrating aptamers with other biomolecules such as enzymes and antibodies could create hybrid systems that harness the strengths of each component, potentially leading to enhanced specificity and multifunctional sensing platforms. Another critical area of development is improving aptamer stability in complex biofluids, where degradation and structural changes can compromise performance. Using aptamers as building blocks for two-dimensional and three-dimensional nanostructures, in addition to their integration with smart, multifunctional nanomaterials such as metal–organic frameworks or flexible polymers, offers a promising approach to preserving their functional conformation and improving signal transduction. A final important advancement in aptamer research is the development of multifunctional aptamers, offering greater versatility, binding multiple targets simultaneously, or switching between binding states in response to stimuli such as light or pH.

Beyond medical applications, aptamers hold great potential in environmental monitoring, where highly selective biosensors could detect pollutants, toxins, and pathogens in water, soil, and air. These advancements will drive the next generation of aptamer-based technologies, making them more robust, scalable, and adaptable to real-world challenges.

## Figures and Tables

**Table 1:** Strategies for aptamer modifications.

Modification	Analyte	Strategy	Properties	Method	Refs.
Nucleic acid terminals	SARS-CoV helicase	Inverted thymidine or biotin	Nuclease resistance	Post-SELEX	[74]
	Interleukin-17A	Pegylation	Nuclease resistance	Post-SELEX	[75]
	33-mer gliadin	5′- thiol, biotin and 6-FAM	Decreased affinity	Post-SELEX	[76]
Sugar ring	HIV-1 reverse transcriptase	2′-deoxy-2′-fluoroarabinonucleotides	Nuclease resistance Stability	Mod-SELEX	[77]
	Thrombin	2′-O,4′-C-methylene-bridged/linked bicyclic ribonucleotides	Binding affinity Nuclease resistance	Mod-SELEX	[78]
Phosphodiester linkage	Thrombin and VEGF165	Phosphorodithioate	Binding affinity Nuclease resistance	Post-SELEX	[79]
Bases	Thrombin	C5-modified thymidine bearing N6-ethyladenine	Binding affinity Nuclease resistance	Mod-SELEX	[78]
	Thrombin	Alkyne	Binding affinity	Mod-SELEX	[80]
	Dengue non-structural protein 1	7-(2-thienyl)imidazo[4,5-b]pyridine (new base)	Binding affinity	Mod-SELEX	[81]
	Human β-defensins	Modified nucleotide triphosphate dAadTP	Binding affinity	Mod-SELEX	[82]
	Thrombin	2′-deoxyuridine analogue bearing an azobenzene moiety at C5-position (dUAzTP)	Photoisomerization	Mod-SELEX	[83]
Spiegelmers	L-CLL2 (Monocyte Chemoattractant Protein-1)	Mirror image	Stability, no immunological response	Post-SELEX	[84,85]
	Bisphenol A	Mirror image	Nuclease resistance	Post-SELEX	[86]
Circular Aptamers	NF-κB	5′ and 3′ ends attached	Stability, nuclease resistance	Genetically encoding	[87]
	Mesenchymal-epithelial transition factor receptors	Hybridizing two monovalent aptamers	Stability, nuclease resistance	Post-SELEX	[88]
Multivalent and Dimerization	Circulating Tumor Cells	Multivalent aptamer functionalized Ag_2_S NDs	Binding affinity	Post-SELEX	[89]
	SARS-CoV-2	Polythymidine (polyT) linker	Binding affinity	Post-SELEX	[90]
Truncation	Tobramycin	Molecular docking	Improved or comparable binding affinity, while reducing costs	Post-SELEX	[91]
	T-2 toxin	Molecular docking	Improved or comparable binding affinity, while reducing costs	Post-SELEX	[71]
	α-Toxin	Molecular docking	Improved or comparable binding affinity, while reducing costs	Post-SELEX	[92]

**Table 2:** Types of signals in aptasensor design, detection strategy, and its application in laboratory settings.

Signal		Strategy		Target	Applications	Ref.
Optical	Fluorescence	Labeled	Capture	Aβ oligomers	Disease diagnosis	[104]
			Sandwich	Carcinoembryonic antigen (CEA), human-α thrombin and prostate-specific antigen (PSA)	Cancer detection	[105]
			Displacement	Aflatoxin B1	Food contamination control	[106]
		Label-free	Capture	Thrombin	Disease diagnosis	[107]
			Displacement	Serotonin	Disease diagnosis	[108]
	Colorimetric	Labeled	Capture	Ochratoxin A	Food contamination control	[109]
			Displacement	microcystin-LR	Environmental monitoring and public health	[110]
			Sandwich	Low-density lipoprotein (LDL)	Cardiovascular disease diagnosis	[111]
		Label-free	Capture	Plasmodium falciparum lactate dehydrogenase (rPfLDH)	Disease diagnosis	[112]
			Displacement	Ochratoxin A	Food contamination control	[113]
	Chemiluminescence	Labeled	Capture	Programmed death ligand-1 (PD-L1) expressing EVs	Cancer detection	[114]
			Displacement	Alpha-fetoprotein detection	Cancer detection	[115]
			Sandwich	CEA	Cancer detection	[116]
		Label-free	Capture	Adenosine triphosphate and chloramphenicol	Food contamination control	[117]
			Displacement	Zearalenone	Food contamination control	[118]
	SERS	Labeled	Capture	Deoxynivalenol	Food contamination control	[119]
			Displacement	Colorectal precancerous lesion biomarkers, hnRNP A1 and S100P	Cancer detection	[120]
			Sandwich	25-hydroxy vitamin D3	Disease diagnosis	[121]
		Label-free	Capture	SARS-CoV-2	Disease diagnosis	[122]
	SPR	Label-free	Capture	C-reactive protein (CRP) and cardiac troponin I (cTn-I)	Disease diagnosis	[123]
Electrical	Electrochemical	Labeled	Capture	CEA	Cancer detection	[124]
			Sandwich	PSA	Cancer detection	[125]
			Displacement	Oestradiol	Disease diagnosis	[126]
		Label free	Capture	Rift Valley fever virus	Disease diagnosis	[127]
			Displacement	Pb^2+^	Environmental monitoring and public health	[128]
	FET	Label-free	Capture	SARS-CoV-2 spike protein	Disease diagnosis	[129]
			Sandwich	Cardiac Troponin I (cTnI)	Disease diagnosis	[130]
	Impedance	Label-free	Capture	Lysozyme	Disease diagnosis	[131]
			Displacement	Thrombin	Disease diagnosis	[132]
Mass sensitive	QCM	Labeled	Displacement	Pb^2+^	Environmental monitoring and public health	[133]
			Sandwich	Hg^2+^	Environmental monitoring and public health	[134]
		Label-free	Capture	SARS-CoV-2 spike	Disease diagnosis	[135]
	SAW (Surface acoustic wave)	Label-free	Capture	Endotoxin from E. Coli	Disease diagnosis	[136]
Micromechanics		Label-free	Capture	PSA	Disease diagnosis	[137]

## Abbreviations

The following abbreviations are used in this manuscript:

SELEX	Systematic Evolution of Ligands by Exponential Enrichment
E-ABs	Electrochemical aptasensors
FRET	Förster Resonance Energy Transfer
CF	Cerebrospinal fluid
FDA	Food and Drug Administration
ISF	Interstitial fluid
CTCs	Circulating tumor cells
PCR	Polymerase chain reaction
MDs	Molecular dynamics
CEA	Carcinoembryonic antigen
PSA	Prostate-specific antigen
LDL	Low-density lipoprotein
rPfLDH	Plasmodium falciparum lactate dehydrogenase
PD-L1	Programmed death ligand-1
EVs	Extracellular vesicles
CRP	C-reactive protein
cTn-I	Cardiac troponin I
MCH	Mercaptohexanol
BBB	Blood–brain barrier
POC	Point-of-care
TDM	Therapeutic drug monitoring
FET	Field-effect transistor
PKU	Phenylketonuria
ATP	Adenosine triphosphate
